# Clinical implications of reduced susceptibility to fluoroquinolones in paediatric *Shigella sonnei* and *Shigella flexneri* infections

**DOI:** 10.1093/jac/dkv400

**Published:** 2015-12-17

**Authors:** Corinne N. Thompson, Nga Tran Vu Thieu, Phat Voong Vinh, Anh Nguyen Duc, Marcel Wolbers, Ha Vinh, James I. Campbell, Dung Tran Thi Ngoc, Nguyen Van Minh Hoang, Tuyen Ha Thanh, Hao Chung The, To Nguyen Thi Nguyen, Nguyen Phu Huong Lan, Christopher M. Parry, Nguyen Van Vinh Chau, Guy Thwaites, Duy Pham Thanh, Stephen Baker

**Affiliations:** 1 Oxford University Clinical Research Unit, Wellcome Trust Major Overseas Programme, The Hospital for Tropical Diseases, Ho Chi Minh City, Vietnam; 2 Centre for Tropical Medicine, Oxford University, Oxford, UK; 3 The London School of Hygiene and Tropical Medicine, London, UK; 4 The Hospital for Tropical Diseases, Ho Chi Minh City, Vietnam; 5 School of Tropical Medicine and Global Health, Nagasaki University, Nagasaki, Japan

## Abstract

**Objectives:**

We aimed to quantify the impact of fluoroquinolone resistance on the clinical outcome of paediatric shigellosis patients treated with fluoroquinolones in southern Vietnam. Such information is important to inform therapeutic management for infections caused by this increasingly drug-resistant pathogen, responsible for high morbidity and mortality in young children globally.

**Methods:**

Clinical information and bacterial isolates were derived from a randomized controlled trial comparing gatifloxacin with ciprofloxacin for the treatment of paediatric shigellosis. Time–kill experiments were performed to evaluate the impact of MIC on the *in vitro* growth of *Shigella* and Cox regression modelling was used to compare clinical outcome between treatments and *Shigella* species.

**Results:**

*Shigella flexneri* patients treated with gatifloxacin had significantly worse outcomes than those treated with ciprofloxacin. However, the MICs of fluoroquinolones were not significantly associated with poorer outcome. The presence of S83L and A87T mutations in the *gyrA* gene significantly increased MICs of fluoroquinolones. Finally, elevated MICs and the presence of the *qnrS* gene allowed *Shigella* to replicate efficiently *in vitro* in high concentrations of ciprofloxacin.

**Conclusions:**

We found that below the CLSI breakpoint, there was no association between MIC and clinical outcome in paediatric shigellosis infections. However, *S. flexneri* patients had worse clinical outcomes when treated with gatifloxacin in this study regardless of MIC. Additionally, *Shigella* harbouring the *qnrS* gene are able to replicate efficiently in high concentrations of ciprofloxacin and we hypothesize that such strains possess a competitive advantage against fluoroquinolone-susceptible strains due to enhanced shedding and transmission.

## Introduction

The Gram-negative bacterial genus *Shigella* are the most common cause of bacillary dysentery globally.^1,2^ Of the four species within the genus, *Shigella flexneri* and *Shigella sonnei* predominate, with *S. sonnei* currently replacing *S. flexneri* as the major species in industrializing regions.^3^ The WHO currently recommends the fluoroquinolone ciprofloxacin as the first-line therapy, with ceftriaxone and pivmecillinam as secondary alternatives.^4^ However, antimicrobial resistance (AMR) within the species is becoming more prevalent and may present a significant challenge for therapeutic management.

The primary target of the fluoroquinolones is the DNA gyrase, a type II topoisomerase essential for DNA replication and transcription.^5^ Mutations in the *gyrA* gene increase the MICs of fluoroquinolones for *Shigella* and other Enterobacteriaceae.^6–9^ Plasmid-mediated quinolone resistance (PMQR) genes can also be acquired, such as the *qnr* genes that encode pentapeptide repeat proteins that bind to and protect the DNA gyrase and topoisomerase from the action of fluoroquinolones.^10^ Complete ciprofloxacin resistance (MIC ≥4 mg/L^11^) has been recently reported in both domestic and imported *S. sonnei* isolates in the USA, Vietnam and elsewhere.^12–14^

The rapid evolution and global dissemination of fluoroquinolone resistance in the Enterobacteriaceae hampers effective treatment and is, therefore, a major threat to human health.^15^ The WHO has explicitly listed fluoroquinolone-resistant *Shigella* as one of its top concerns in the current international focus on AMR.^15^ AMR can lead to inappropriate choice of antimicrobial for initial therapy and may force clinicians to choose more toxic or more expensive antimicrobials.^16^ Furthermore, patients infected with fluoroquinolone-resistant *Campylobacter* and *Salmonella* infections in the USA have been shown to have a longer duration of diarrhoea compared with those infected with fluoroquinolone-susceptible strains.^17,18^ Although one study from Vietnam suggested a correlation between increasing AMR levels in *S. sonnei* and clinical severity,^19^ no rigorous evaluation of the impact of fluoroquinolone resistance or presence of *gyrA* mutations on clinical outcome of *Shigella* infections has been performed.

Here, we aimed to quantify the effect of fluoroquinolone choice, fluoroquinolone susceptibility and presence of *gyrA* mutations on fever clearance time (FCT) and total duration of illness in children with *S. flexneri* and *S. sonnei* infections in Vietnam. Additionally, we sought to compare the severity and AMR profiles between *Shigella* species as well as investigate the effect of elevated MIC and *gyrA* mutations on the *in vitro* activity of *Shigella.* Understanding the dynamics of increasing MICs of commonly used fluoroquinolones and clinical patient outcome in industrializing locations is important as it allows clinicians to be better informed when prescribing therapies for what can often be severe infections in young children.

## Methods

### Patient population

The source data for this study was a randomized controlled trial. The protocol (including justification for use of gatifloxacin) and results for this trial have been described previously in detail.^20^ Briefly, 500 children were enrolled into an open-label, randomized clinical trial comparing 3 day regimens of gatifloxacin (10 mg/kg/day orally in one dose) and ciprofloxacin (30 mg/kg/day orally in two doses) for the treatment of shigellosis in southern Vietnam. Children were enrolled between 2006 and 2009 at the Hospital for Tropical Diseases in Ho Chi Minh City and at Huu Nghi Hospital in Dong Thap province. Inclusion criteria included age <15 years and a history of bloody or mucoid stools in the 72 h prior to admission to hospital. Exclusion criteria included severe infection (shock, jaundice and extensive gastrointestinal bleeding), known treatment with a fluoroquinolone during the episode and concomitant infection requiring antimicrobial therapy.

### Study procedures

Daily case report forms detailing clinical presentation were administered for each patient during the period of hospitalization. A case report form was also administered at a follow-up visit that occurred 7 days after discharge. Clinical failure was defined as fever (≥37.8°C) or the persistence of any signs or symptoms after 120 h of start of treatment (vomiting, abdominal pain or tenesmus with/without three or more loose stools with/without blood and/or mucus). Total duration of symptoms was defined as the time from admission until cessation of all listed symptoms. Microbiological failure was defined as a positive stool culture for the original infecting pathogen after day 3 of the antimicrobial therapy. FCT was defined as the time from admission until temperature was ≤37.8°C for ≥48 h.

Stool samples were collected on admission and standard microbiological techniques were employed to identify *Shigella* and *Salmonella* isolates.^20^ Antimicrobial susceptibility testing was performed by disc diffusion following methods prescribed by the CLSI.^11^ MICs were calculated by Etest as per the manufacturer's instructions (AB Biodisk, Sweden). Strains that were identified as resistant to ceftriaxone were subjected to further phenotypic tests to confirm ESBL production using discs containing only cefotaxime (30 μg) and both cefotaxime and ceftazidime combined with clavulanic acid (10 μg), according to current CLSI guidelines.^11^

### Genomic DNA extraction, PCR and sequencing

Genomic DNA was purified using the Wizard genomic DNA extraction kit (Promega, USA) as recommended by the manufacturer. Extracted DNA was subjected to PCR targeting known mutation regions on *gyrA* and *parC* genes and the PMQR genes *qnrA*, *qnrB*, *qnrC*, *qnrS*, *aac(6′)-Ib-cr* and *qepA*. Primer sequences were *GyrA*_F: 5′-CGACCTTGCGAGAGAAAT-3′, *GyrA*_R: 5′-GTTCCATCAGCCCTTCAA-3′,^21^ *ParC*_F: 5′-AAACCTGTTCAGCGCCGCATT-3′ and *ParC*_R: 5′-GTGGTGCCGTTAAGCAAA-3′.^22^ The primers for the PMQR genes were as previously published.^23–25^ *Taq* DNA polymerase supplied by Bioline (UK) was used for the amplifications. Concentrations of reagents were as recommended by the manufacturers. PCR amplifications were performed under the following conditions: 1 cycle of 95°C for 5 min followed by 35 cycles of 95°C for 30 s, 55°C for 30 s and 72°C for 1 min. PCR amplicons were sequenced using an ABI 3700 system (ABI, USA) and sequencing reactions were prepared as recommended by the manufacturer. Resulting sequences were analysed using Bioedit software.

### Time–kill analyses

One isolate from each mutation group was selected for *in vitro* time–kill experiments, all were *S. flexneri* isolates: (i) no *gyrA* mutation, ciprofloxacin MIC 0.023 mg/L and gatifloxacin MIC 0.023 mg/L; (ii) *gyrA* mutation A87T, ciprofloxacin MIC 0.094 mg/L and gatifloxacin MIC 0.125 mg/L; (iii) *gyrA* mutation S83L, ciprofloxacin MIC 0.19 mg/L and gatifloxacin MIC 0.19 mg/L; and (iv) *gyrA* mutation S83L, ciprofloxacin MIC 8 mg/L and gatifloxacin MIC 6 mg/L with *qnrS* gene. Strains were grown overnight in Mueller–Hinton (MH) broth. *S. flexneri* isolates were chosen due to the larger range of MICs of fluoroquinolones compared with those for *S. sonnei.* The bacterial cultures were diluted 1 : 1000 into 10 mL of fresh MH broth. The inoculation was incubated at 37°C with a circular agitation speed of 150 rpm for 1.5 h. The cultures were mixed with either ciprofloxacin or gatifloxacin, which had been prepared in MH broth to achieve the final volume of 20 mL of the desired concentration of drugs. Controls for each mutant were identical cultures but without supplementary antimicrobials. Bacterial cells were counted at time 0 and 30 min, 1 h, 2 h, 4 h, 6 h, 12 h and 24 h after incubation with antimicrobials. The experiment was performed in replicates of nine for each selected isolate. The limit of detection was 10 cfu/mL.^26^

### Statistical analyses

All data were analysed in STATA v13 (TX, USA). Plots were made in R (R Foundation for Statistical Computing, Austria) using the ggplot2 package.^27^ Continuous data were compared between groups using the Kruskal–Wallis test. Categorical group data were compared using *χ*^2^ or Fisher's exact test. For MICs that were recorded as ‘greater than X’ or ‘less than X’, these values were converted into 2X and X/2, respectively. Logistic regression was used to evaluate the relationship between treatment arm, MICs and overall failure, with interaction between drug and MIC evaluated through the likelihood ratio test. We analysed the time to event endpoints of FCT and total duration of symptoms using Cox regression models. Interaction between species and treatment arm as well as MIC and treatment within species were evaluated using the likelihood ratio test. Age was included *a priori* as a covariate in all models.

### Ethics approval

This study was approved by the institutional ethics review boards of the Hospital for Tropical Diseases and Huu Nghi Hospital and the Oxford Tropical Research Ethics Committee (OxTREC: 010-06). Written informed consent from the parent or guardian was required for enrolment into the trial.

## Results

### Baseline clinical and demographic characteristics

Of the 500 children enrolled, 6 withdrew after randomization, leaving 494 for analysis. A total of 107/494 (22%) enrolled trial patients were stool culture positive for *Shigella* spp. Of these, 72 (67%) were *S. sonnei*, 33 (31%) were *S. flexneri* and 2 (2%) were *Shigella boydii.* As shown in Table 1, *S. sonnei* patients were slightly younger (median: 30 months, IQR: 20–43) than *S. flexneri* patients (median: 36 months, IQR: 22–60) and were more likely to report a greater number of mucoid stools in the first 24 h after admission compared with *S. flexneri* patients. *S. sonnei* patients also had slightly elevated white cell counts (median 13.5 × 10^9^/L, IQR: 10.7–16.9) compared with *S. flexneri* patients (median: 10.2 × 10^9^/L, IQR: 7.3–16.4). *S. flexneri* patients, however, were more likely to report abdominal pain (91%) prior to admission than *S. sonnei* patients (72%).

**Table 1.** Baseline demographic and clinical characteristics of all patients, *S. sonnei* patients and *S. flexneri* patients

Characteristic	All patients, *n* = 494	*S. sonnei* patients, *n* = 72	*S. flexneri* patients, *n* = 33	*P*^a^
Site, *n* (%)				
Ho Chi Minh City	194 (39.3)	48 (66.7)	20 (60.6)	0.546
Dong Thap	300 (60.7)	24 (33.3)	13 (39.4)	
Study drug, *n* (%)				
ciprofloxacin	245 (49.6)	34 (47.2)	12 (36.4)	0.298
gatifloxacin	248 (50.2)	38 (52.8)	21 (63.6)	
Age (months), median (IQR)	19 (10.5–32)	30 (20–43)	36 (22–60)	0.062
Male, *n* (%)	291 (58.9)	40 (55.6)	14 (42.4)	0.211
Nutritional status, *n* (%)				
overweight	4 (0.8)	1 (1.4)	0 (0)	0.089
normal	363 (73.5)	60 (83.3)	26 (78.8)	
malnutrition I	93 (18.8)	10 (13.9)	3 (9.1)	
malnutrition II	29 (5.9)	1 (1.4)	4 (12.1)	
malnutrition III	5 (1.0)	0 (0)	0 (0)	
Prior to admission				
illness duration (h), median (IQR)	24 (16–48)	20 (12–33)	19 (12–24)	0.785
fever (≥37.8°C), *n* (%)	429 (87.4)	68 (94.4)	33 (100)	0.167
history of febrile convulsions, *n* (%)	40 (8.1)	6 (8.3)	2 (6.1)	1.000
history of diarrhoea with blood, *n* (%)	210 (42.5)	24 (33.3)	11 (33.3)	1.000
history of mucoid diarrhoea without blood, *n* (%)	284 (57.5)	48 (66.7)	22 (66.7)	1.000
vomiting, *n* (%)	204 (41.3)	34 (47.2)	16 (48.5)	0.904
abdominal pain, *n* (%)	365/492 (74.2)	52 (72.2)	30 (90.9)	0.041
tenesmus, *n* (%)	339/490 (69.2)	45/71 (63.4)	22 (66.7)	0.745
Within 24 h of admission				
mucoid diarrhoea without blood, *n* (%)	370 (74.9)	59 (81.9)	22 (66.7)	0.083
number of mucoid stools/24 h, median (IQR)	3 (0–6)	4 (2–10)	2 (0–5)	0.028
diarrhoea with blood, *n* (%)	445 (90.1)	70 (97.2)	33 (100)	0.334
number of bloody stools/24 h, median (IQR)	1 (1–5)	3 (1–6)	3 (1–9)	0.187
maximum number of episodes/24 h, median (IQR)	6 (3–10)	8 (3–11)	8 (4–10)	0.806
white blood cells in stool (cells/HPF), *n* (%)				
0	214/479 (44.7)	14/68 (20.6)	8/32 (25.0)	0.754
1–10	79/479 (16.5)	11/68 (16.2)	4/32 (12.5)	
11–20	42/479 (8.8)	5/68 (7.4)	4/32 (12.5)	
21–30	104/479 (21.7)	26/68 (38.2)	9/32 (28.1)	
>30	40/479 (8.4)	12/68 (17.6)	7/32 (21.9)	
white cell count ×10^9^/L, median (IQR)	11.3 (8–14.9)	13.5 (10.7–16.9)	10.2 (7.3–16.4)	0.071

HPF, high power field.

^a^*P* value comparing *S. sonnei* and *S. flexneri* by Kruskal–Wallis test for continuous data or χ^2^/Fisher's exact test for categorical data.

### Antimicrobial susceptibility

**Figure 1.**
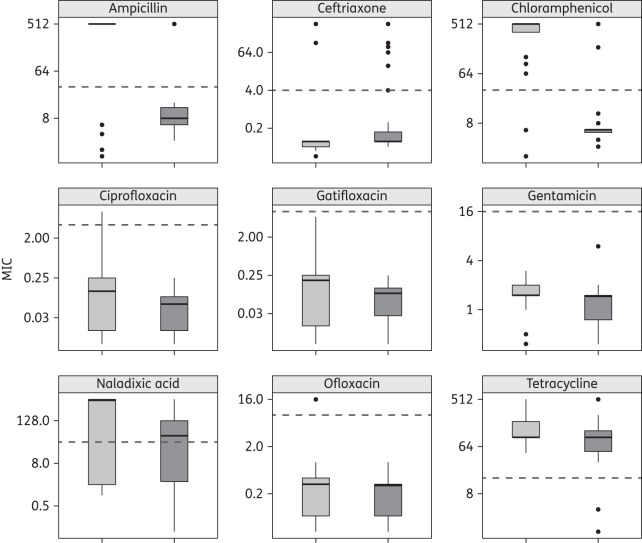
MICs (mg/L) of a range of antimicrobials for the *Shigella* isolates in this study (log_2_ scale). Box plots show the median (black line across each box) and the 5th and 95th percentiles. MICs for *S. sonnei* are shown in dark grey and MICs for *S. flexneri* are in light grey. The broken line in each plot represents the current CLSI breakpoint for resistance.^11^

As shown in Table 2, *S. flexneri* isolates were more likely to have higher MICs against the fluoroquinolones (with the exception of ofloxacin) compared to *S. sonnei.* In fact, *S. flexneri* had significantly higher MICs of all tested antimicrobials with the exception of ceftriaxone, for which *S. sonnei* had significantly elevated MICs over *S. flexneri* (*P* < 0.001, Kruskal–Wallis test) (Figure 1). Overall, *S. flexneri* isolates were more likely to be MDR (defined here as non-susceptible to more than three antimicrobial classes) (28/33, 85%) compared with *S. sonnei* (22/72, 31%) (*P* < 0.001, Fisher's exact test) while *S. sonnei* isolates were more likely to exhibit an ESBL phenotype (14/72, 19%) than *S. flexneri* (1/33, 3%) (*P* = 0.033, Fisher's exact test) (Figure S1, available as Supplementary data at *JAC* Online). Finally, 3/33 (9%) of the tested *S. flexneri* isolates and 1/72 (1%) of tested *S. sonnei* isolates were PCR amplification positive for the *qnrS* gene. No *parC* mutations, *qnrA*, *qnrB*, *aac(6′)-Ib-cr* or *qepA* genes were identified within any of the *Shigella* isolates.

**Table 2.** Comparison of MICs of fluoroquinolones for *S. sonnei* and *S. flexneri* isolates

Antimicrobial	*S. sonnei* MIC (mg/L), *n* = 72	*S. flexneri* MIC (mg/L), *n* = 33	*P*^a^
Nalidixic acid			
median (range)	48 (0.09–512)	512 (1–512)	0.046
geometric mean	35.3	68.4	
Ciprofloxacin			
median (range)	0.064 (0.01–0.25)	0.125 (0.01–8)	0.011
geometric mean	0.05	0.08	
Gatifloxacin			
median (range)	0.094 (0.01–0.25)	0.19 (0.01–6)	0.008
geometric mean	0.06	0.09	
Ofloxacin			
median (range)	0.38 (0.05–1)	0.38 (0.05–16)	0.135
geometric mean	0.25	0.3	

^a^*P* value comparing MIC between species by Kruskal–Wallis test.

The MICs of fluoroquinolones were highly correlated for both *S. sonnei* and *S. flexneri* (*P* < 0.001 for all correlations). When normalized to the mean of the current CLSI resistance breakpoint (ciprofloxacin: ≥4 mg/L, gatifloxacin: ≥8 mg/L),^11^ both *S. flexneri* and *S. sonnei* had higher relative median log_2_ MICs of ciprofloxacin than of gatifloxacin (*P* < 0.001 in both cases, Kruskal–Wallis test of *z*-score) (Figure S2). Furthermore, the presence of a single *gyrA* mutation and/or the *qnrS* gene dramatically increased the MICs of fluoroquinolones for both *S. sonnei* and *S. flexneri* (Table 3 and Figure S3).

**Table 3.** MICs of fluoroquinolones for *S. sonnei* and *S. flexneri* by *gyrA* mutation and *qnrS* gene, median (range)

Antimicrobial	*S. sonnei* MIC (mg/L)				*S. flexneri* MIC (mg/L)			
	no mutation, *n* = 19	A87T, *n* = 40	S83L, *n* = 9	S83L/*qnrS*, *n* = 1	no mutation, *n* = 11	S83L, *n* = 16	*qnrS*, *n* = 1	S83L/*qnrS*, *n* = 2
Nalidixic acid	1.50 (0.09–3.0)	64 (32–512)	96 (64–512)	512	2.0 (1.0–4.0)	512 (512–512)	1.50	512 (512–512)
Ciprofloxacin	0.01 (0.01–0.02)	0.06 (0.05–0.13)	0.19 (0.13–0.25)	0.25	0.02 (0.01–0.05)	0.19 (0.09–0.25)	0.01	4.13 (0.25–8.0)
Gatifloxacin	0.01 (0.01–0.13)	0.09 (0.06–0.13)	0.19 (0.13–0.25)	0.19	0.01 (0.01–0.06)	0.19 (0.13–0.38)	0.01	3.13 (0.25–6.0)
Ofloxacin	0.06 (0.05–0.09)	0.38 (0.25–0.50)	0.75 (0.5–1.0)	0.75	0.09 (0.05–0.19)	0.5 (0.38–1.0)	0.05	8.25 (0.5–16)

All pairwise comparisons within species across antimicrobials were statistically significantly different by Kruskal–Wallis tests (all *P* < 0.001) and all pairwise comparisons of A87T and S83L (regardless of *qnrS*) in *S. sonnei* were statistically significantly different (*P* < 0.001), except for A87T versus S83L for nalidixic acid (*P* = 0.03), by Kruskal–Wallis test.

### Time–kill curves

**Figure 2.**
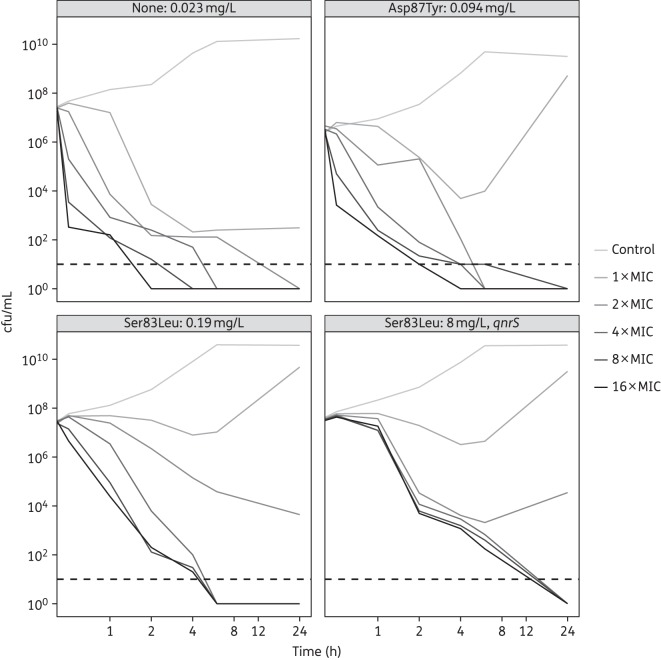
Time–kill curves of *S. flexneri gyrA* (*qnrS*) genotypes on exposure to increasing concentrations of ciprofloxacin. Plots showing the mean time–kills of *S. flexneri* isolates grown with increasing concentrations of ciprofloxacin based on the MIC for the original isolate at different timepoints post-inoculation (log_2_ scale). The *S. flexneri* isolates are: (a) no *gyrA* mutation, ciprofloxacin MIC = 0.023 mg/L; (b) *gyrA* mutation A87T, ciprofloxacin MIC = 0.094 mg/L; (c) *gyrA* mutation S83L, ciprofloxacin MIC = 0.19 mg/L; and (d) *gyrA* mutation S83L, ciprofloxacin MIC = 8 mg/L with the *qnrS* gene. The broken line represents the limit of detection for the assay (10 cfu/mL).

Mutations in *gyrA*, the presence of *qnrS* and elevated MICs of ciprofloxacin substantially increased the ability of *S. flexneri* to replicate in the presence of ciprofloxacin (Figure 2). The *S. flexneri* strain lacking a *gyrA* mutation with a low MIC of ciprofloxacin was rapidly killed by the antimicrobial (Figure 2). While the isolate with the *gyrA* mutation A87T (Figure 2) was still effectively killed at higher concentrations of ciprofloxacin, bacterial growth remained elevated at lower concentrations compared with the *S. flexneri* without a *gyrA* mutation. The S83L mutation (Figure 2) reduced the bactericidal activity of ciprofloxacin even further, with cfu/mL reduced by >5 logs at high concentrations. Finally, the *S. flexneri* isolate with an S83L *gyrA* mutation, *qnrS* gene and MIC of ciprofloxacin of 8 mg/L (Figure 2) was able to grow in the presence of all tested concentrations of ciprofloxacin during the first hour post-inoculation, with growth falling by 2 logs only within the second hour post-inoculation. Further, the concentration-dependent killing effect was lost with the *qnrS* mutant, as the 2× to 16× MIC curves exhibited almost identical profiles. The same pattern was observed with the corresponding gatifloxacin time–kill curve (data not shown).

### Clinical outcome in shigellosis with fluoroquinolone therapy

Overall, there were 8 (7.6%) failures among the 105 *Shigella* cases, all of which were *S. sonnei* (8/72, 11%). Two of these cases were microbiological failures and six were classified as clinical failures. Overall failure amongst the *S. sonnei* isolates was not significantly associated with either treatment drug (OR: 0.5, 95% CI: 0.1–2.3, *P* = 0.37) or MIC of nalidixic acid, ciprofloxacin, gatifloxacin or ofloxacin. Additionally, although 5/8 (63%) failures had the A87T *gyrA* mutation and 1/8 (13%) had the S83L *gyrA* mutation, there was no significant effect of a *gyrA* mutation on failure (OR: 1.03, 95% CI: 0.3–3.3, *P* = 0.96).

**Figure 3.**
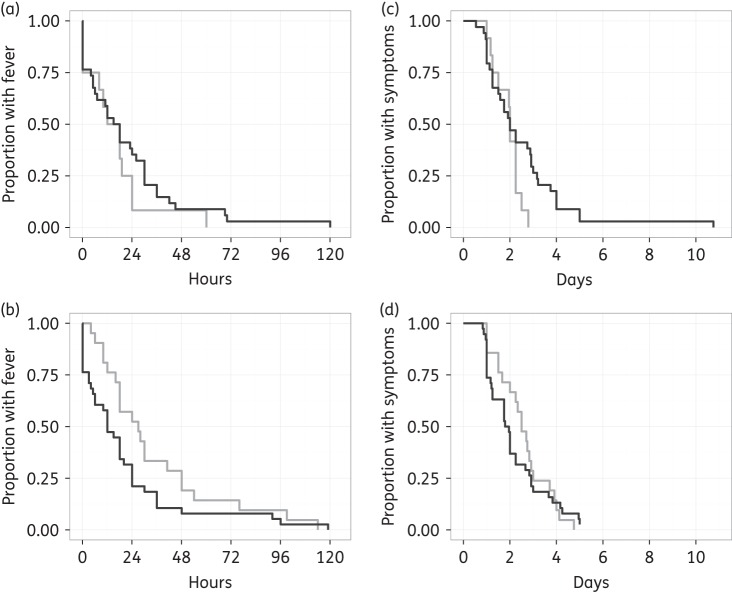
Clinical outcome comparison between *S. sonnei* and *S. flexneri* infections treated with fluoroquinolones. Unadjusted Kaplan–Meier plots showing FCT in hours in patients treated with (a) ciprofloxacin (*n* = 46) and (b) gatifloxacin (*n* = 59). Total duration of symptoms in days is shown in patients treated with (c) ciprofloxacin and (d) gatifloxacin. *S. sonnei* are shown in dark grey and *S. flexneri* are shown in light grey.

Overall, the FCTs of *S. sonnei* and *S. flexneri* were not significantly different (HR: 1.2, 95% CI: 0.8–1.8, *P* = 0.47). There was no difference in FCT between the two species in the ciprofloxacin arm (HR: 0.73, 95% CI: 0.4–1.4, *P* = 0.355) and although *S. flexneri* patients had moderately longer FCTs in the gatifloxacin arm (median: 27 h, IQR: 16–48) compared with *S. sonnei* (median: 12 h, IQR: 3–24), the difference was not statistically significant after controlling for age (HR: 1.52, 95% CI: 0.9–2.7, *P* = 0.13) (Figure 3a and b). Notably, the FCTs of *S. flexneri* treated with gatifloxacin (median: 27 h, IQR: 16–48) were significantly longer than for *S. flexneri* patients treated with ciprofloxacin (median: 15, IQR: 4–22) (HR: 0.40, 95% CI: 0.2–0.9, *P* = 0.02). Similar patterns were observed for total duration of symptoms (Figure 3c and d), as *S. flexneri* patients treated with gatifloxacin (median: 60 h, IQR: 4–72) had significantly longer duration of symptoms compared with *S. flexneri* patients treated with ciprofloxacin (median: 48 h, IQR: 33–54) (HR: 0.39, 95% CI: 0.2–0.9, *P* = 0.02). No difference in total duration of symptoms was detected between the treatment arms for *S. sonnei* patients. The presence of *gyrA* mutations did not have a significant effect on either FCT or total duration of symptoms (*P* > 0.05 for both comparisons).

**Figure 4.**
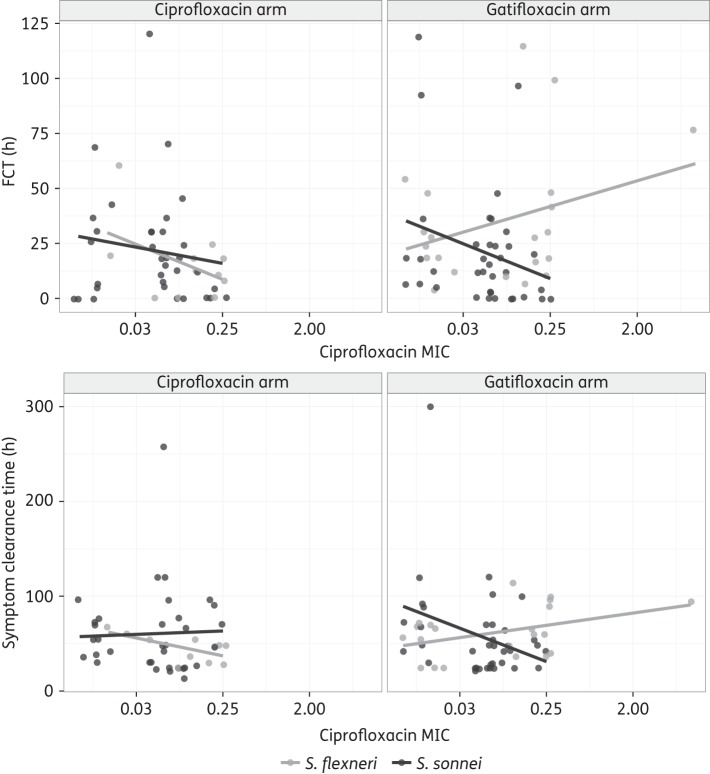
Effect of MIC of ciprofloxacin (mg/L) on clinical outcome for *S. sonnei* and *S. flexneri* infections. Associations between ciprofloxacin and FCT (top two plots) and symptom clearance time (bottom two plots) are shown. Patients treated with ciprofloxacin are shown on the left and patients treated with gatifloxacin are on the right. Patients infected with *S. sonnei* are shown in dark grey and patients infected with *S. flexneri* are in light grey. The lines represent the best-fit linear model for each set of patients.

When evaluating the effect of MICs on FCT and duration of symptoms, we found a significant interaction between treatment arm and gatifloxacin and ciprofloxacin MICs for *S. flexneri* patients (*P* < 0.03 in all comparisons). Although increasing ciprofloxacin MICs appeared to be associated with increased FCT and symptom duration in *S. flexneri* patients treated with gatifloxacin (Figure 4), these trends were not statistically significant (effect of log_2_ ciprofloxacin MIC on (i) FCT: HR: 0.92 (95% CI: 0.8–1.1, *P* = 0.33); and (ii) symptom duration: HR: 0.86 (95% CI: 0.7–1.1, *P* = 0.15). This pattern was comparable for the gatifloxacin MIC (data not shown). Finally, there was no significant effect of MIC of either antimicrobial in the ciprofloxacin treatment arm or amongst *S. sonnei* patients in either treatment arm.

## Discussion

The bacterial genus *Shigella* are a considerable public health problem, responsible for >7 million disability-adjusted life years and >100 000 deaths annually, which are mainly concentrated in children living in developing countries.^1,2,28,29^ The current drug of choice for treating *Shigella* infections is ciprofloxacin. However, fluoroquinolone resistance is threatening to make the management of this diarrhoeal pathogen even more challenging.^15^ Here, we aimed to quantify the impact of treatment choice, fluoroquinolone MIC and presence of *gyrA* mutations on clinical outcome of paediatric *S. flexneri* and *S. sonnei* patients treated with fluoroquinolones. We additionally evaluated the ability of *Shigella* strains of varying resistance profiles to grow in the presence of ciprofloxacin through time–kill experiments rather than single timepoint MIC testing.

While we hypothesized that poorer clinical outcome was associated with higher fluoroquinolone MIC and observed this trend in our data, we were unable to conclude that such relationships were statistically significant. This is likely due to small numbers of patients and relatively low MICs for isolates collected during the time period (2006–09). Mutations in *gyrA* were not found to be associated with an inferior clinical outcome in either species, although S83L and A87T mutations did result in isolates with higher MICs of fluoroquinolones. We hypothesize that fluoroquinolone MICs may play a greater role in clinical outcome above the CLSI breakpoint as we have shown conclusively that below the current breakpoints, MIC does not have a significant impact on patient outcome. Therefore, we surmise that the current CLSI breakpoints for fluoroquinolones are appropriate for *Shigella* therapy.

We identified differences in clinical response between both the *Shigella* species and treatment arms. *S. flexneri* patients had significantly longer FCTs and duration of symptoms when treated with gatifloxacin compared with ciprofloxacin. Gatifloxacin is presumed to be a more efficacious drug than ciprofloxacin,^30^ yet *S. flexneri* isolates were more likely to have higher MICs of ciprofloxacin (relative to the CLSI breakpoint) compared with gatifloxacin. In the absence of additional data assessing the penetration of gatifloxacin into the epithelial cells lining the gastrointestinal tract, we hypothesize this difference between antimicrobials in *S. flexneri* patients may be due to more effective killing of the local commensal gut microbiota by gatifloxacin, which may allow *Shigella* to more efficiently invade the gut tissue.^31–33^ Further investigation into the effects of fluoroquinolones on the gut microbiota are required.

The time–kill experiments clearly demonstrate that an elevated MIC, and most notably presence of the combination of a *qnrS* gene and a *gyrA* mutation, allows *Shigella* to replicate efficiently in high concentrations of fluoroquinolone. We hypothesize that such enhanced persistence in the gastrointestinal tract during therapy endows fluoroquinolone-resistant *Shigella* with a competitive advantage over fluoroquinolone-susceptible strains. AMR *Shigella* are not only more likely to survive therapy but also to be shed and further transmitted in the community, over time replacing the fluoroquinolone-susceptible strains. For example, the acquisition of chloramphenicol resistance is understood to have afforded certain clones of *Salmonella enterica* serovar Typhimurium the ability to replace susceptible strains in sub-Saharan Africa over several years.^34^ Furthermore, there is limited evidence that suggests that an elevated MIC of nalidixic acid lengthens the duration of excretion of *Shigella.*^35^

The time–kill results also suggest that acquisition of *qnrS* in addition to an S83L mutation in *gyrA* may lead to a loss of concentration-dependent killing by fluoroquinolones. This has been observed previously in *Escherichia coli* with the *qnrS* gene^36^ and may relate to the mechanism of action of *qnrS* whereby peptides bind to the DNA gyrase and block fluoroquinolone activity. Although higher concentrations of fluoroquinolones are generally preferred in therapy to reduce the likelihood of development of resistant strains,^37,38^ *qnrS* mutants may not respond to high doses given our time–kill observations. Therefore, combination therapy in patients with *qnrS*-positive *Shigella* infections could be warranted. Further pharmacodynamic work on *qnrS* mutants in the Enterobacteriaceae is clearly required.

This study has some limitations. First, FCT and duration of symptoms may not be the most appropriate measures of clinical outcome. We therefore may have under- or overestimated the effect of fluoroquinolone resistance on patient outcome. Second, we had a limited range of fluoroquinolone MICs and a limited number of treatment failures, which made it difficult to better evaluate trends in the isolates with elevated MICs or in those that failed treatment. It will be important to repeat this work with contemporary strains as higher levels of fluoroquinolone resistance will permit such an investigation.^39^ Furthermore, collection of longitudinal stool samples after treatment is warranted to understand the effect of resistance on excretion duration. Nonetheless, the work presented here offers the first rigorous analysis of fluoroquinolone resistance on patient outcome to our knowledge and fills an important gap in the knowledge of this increasingly antimicrobial-resistant pathogen.

We conclude that below the CLSI breakpoint, MICs of fluoroquinolones do not strongly impact patient outcome in shigellosis. Therefore, the current CLSI breakpoints are warranted for *Shigella* infections. However, our data suggest that the choice of fluoroquinolone is important in the management of *Shigella* infections, as *S. flexneri* patients treated with gatifloxacin had poorer outcomes compared with those treated with ciprofloxacin. Further, we demonstrate that *qnrS*-harbouring *Shigella* are able to grow effectively *in vitro* at high concentrations of ciprofloxacin and hypothesize that fluoroquinolone-resistant strains outcompete susceptible strains, as they are maintained during therapy, shed and therefore more likely to be transmitted in the community. Continued evaluation of the impact of fluoroquinolone resistance on the clinical outcome of *Shigella* patients over time is critical to help inform clinical treatment decisions for diarrhoeal infections.

## Funding

This work was supported by the Wellcome Trust of the UK, through core funding (089276/2/09/2). S. B. is a Sir Henry Dale Fellow, jointly funded by the Wellcome Trust and the Royal Society (100087/Z/12/Z).

## Transparency declarations

None to declare.

## Supplementary data

Figures S1 to S3 are available as Supplementary data at *JAC* Online (http://jac.oxfordjournals.org/).

Supplementary Data
