# Supplementary material for: Clinical implications of reduced susceptibility to fluoroquinolones in paediatric Shigella sonnei and Shigella flexneri infections
Source: J Antimicrob Chemother. 2015 Dec 17;71(3):807–15. doi: 10.1093/jac/dkv400 (PMC4743702; doi:10.1093/jac/dkv400)
Supplement: Supplementary Data [file supp_71_3_807__index.html]

Clinical implications of reduced susceptibility to fluoroquinolones in paediatric Shigella sonnei and Shigella flexneri infections — Clinical implications of reduced susceptibility to fluoroquinolones in paediatric Shigella sonnei and Shigella flexneri infections — Supplementary Data 

# Clinical implications of reduced susceptibility to fluoroquinolones in paediatric *Shigella sonnei* and *Shigella flexneri* infections

## Supplementary Data

Supplementary Data

- Supplementary Data - Docx file
