# Supplementary material for: Clinical implications of reduced susceptibility to fluoroquinolones in paediatric Shigella sonnei and Shigella flexneri infections
Source: J Antimicrob Chemother. 2015 Dec 17;71(3):807–15. doi: 10.1093/jac/dkv400 (PMC4743702; doi:10.1093/jac/dkv400)
Supplement: Supplementary Data [file supp_dkv400_dkv400supp.docx]

**Supplementary data**

**Figure S1.** The proportions of *S. flexneri* and *S. sonnei* isolates exhibiting antimicrobial resistance. A bar chart showing the proportion of isolates fully resistant to each antimicrobial according to 2014 CLSI guidelines.^11^ *S. sonnei* are shown in dark grey and *S. flexneri* in light grey. AMP: ampicillin; CHL: chloramphenicol; CIP: ciprofloxacin; CRO: ceftriaxone; GAT: gatifloxacin; GEN: gentamycin; NAL: nalidixic acid; OFX: ofloxacin; SXT: cotrimoxazole; TET: tetracycline; MDR: multidrug resistant (defined as resistance to ampicillin, chloramphenicol and cotrimoxazole); ESBL: extended-spectrum beta lactamase phenotype.

**Figure S2.** Distribution of MICs of *S. sonnei* and *S. flexneri* against ciprofloxacin and gatifloxacin relative to the CLSI resistance breakpoint. Density plots showing the Z-scores of log2MICs (mg/L) with the mean cantered on the CLSI breakpoint (ciprofloxacin: 1mg/L, gatifloxacin: 8mg/L). Gatifloxacin MICs are shown in the dashed line and ciprofloxacin MICs are shown in the solid line. Plots are separated by species: (A) *S. flexneri* and (B) *S. sonnei*. CIP: ciprofloxacin; GAT: gatifloxacin.

**Figure S3.** The distribution of MICs of *gyrA* mutations against four fluoroquinolones. Density plots showing MICs (mg/L) to the (fluoro)quinolones for each *gyrA* mutation. Isolates without a *gyrA* mutation are shown in light grey, isolates with the A87T mutation are shown in dark grey and isolates with the S83L mutation are shown in black. The MICs of (A) nalidixic acid (B) gatifloxacin (C) ciprofloxacin and (D) ofloxacin are shown on a log2 scale.
